# The relationship between hand osteoarthritis and serum leptin concentration in participants of the Third National Health and Nutrition Examination Survey

**DOI:** 10.1186/ar3864

**Published:** 2012-05-31

**Authors:** Mei Massengale, William M Reichmann, Elena Losina, Daniel H Solomon, Jeffrey N Katz

**Affiliations:** 1 Division of Rheumatology, Brigham and Women's Hospital, 75 Francis Street, Boston, MA 02115, USA; 2 Harvard Medical School, Longwood Avenue, Boston, MA 02115, USA; 3 Department of Orthopedic Surgery, Brigham and Women's Hospital, 75 Francis Street, Boston, MA 02115, USA; 4 Orthopaedic and Arthritis Center for Outcomes Research, Brigham and Women's Hospital, 75 Francis Street, Boston, MA 02115, USA; 5 Department of Biostatistics, Boston University School of Public Health, 715 Albany Street, Boston, MA 02118, USA; 6 Division of Pharmacoepidemiology and Pharmacoeconomics, Brigham and Women's Hospital/Harvard Medical School, 75 Francis Street, Boston, MA 02115, USA; 7 Department of Epidemiology, Harvard School of Public Health, 677 Huntington Avenue, Boston, MA 02115, USA

## Abstract

**Introduction:**

Leptin has been suspected to contribute to the development of osteoarthritis (OA). However, this hypothesis has not been tested in large-scale hand OA cohorts. Our study aimed to determine whether there is a cross-sectional relationship between serum leptin levels and hand OA in a population-based sample of US adults.

**Method:**

We used the Third National Health and Nutrition Examination Survey (NHANES III), a national cross-sectional population-based survey, to study the relationship between hand OA and serum leptin concentration. We applied previously established classification criteria for hand OA. Patients with rheumatoid arthritis were excluded. Potential confounders included sex, body mass index, the presence of polyarticular OA, diabetes, and total cholesterol. We estimated unadjusted mean leptin concentration by hand OA status and by all confounders. We further developed a linear regression model to assess mean leptin levels, adjusted for appropriate confounders.

**Results:**

Of 2,477 subjects in the NHANES III sample that had a hand examination and did not have rheumatoid arthritis, 1,056 (42.6%) had a leptin measurement and were included in the analysis. Subjects with and without leptin measurement had similar demographic characteristics. We did not find any significant differences in mean serum leptin levels in subjects with symptomatic hand OA (7.38 ng/ml in males (95% confidence interval (CI) = 5.31, 9.46) and 21.55 ng/ml in females (95% CI = 17.08, 26.02)), asymptomatic hand OA (6.69 ng/ml in males (95% CI = 5.19, 8.18) and 17.09 ng/ml in females (95% CI = 15.00, 19.18)), and no hand OA (8.22 ng/ml in males (95% CI = 7.47, 8.97) and 20.77 ng/ml in females (95% CI = 18.01, 23.53)) in the unadjusted analysis. In a multivariable linear regression model that included variables of hand OA status, age, race/ethnicity, and obesity status, we found no statistically significant association between serum leptin and hand OA status.

**Conclusions:**

In this cross-sectional study of a large representative US cohort, we did not find any evidence to support the hypothesis that serum leptin is associated with hand OA.

## Introduction

Hand osteoarthritis (OA) is a common joint disease worldwide, resulting in weakened grip strength and significant functional disability [1]. As nonweight-bearing joints, the hand is a useful target for the study of nonmechanical risk factors for OA pathogenesis [2-10].

Obesity has been observed to have associations with OA development in nonweight-bearing joints as well as in weight-bearing joints [11-14]. Adipokine hormones such as leptin have long been thought to contribute to OA pathogenesis directly, independent of the mechanical effect of obesity [3,4,11,15]. Leptin, a small polypeptide, is predominantly produced in white adipose tissue and regulates food intake and energy expenditure. Leptin has also been increasingly recognized to play a role in inflammation, angiogenesis, as well as cartilage and bone metabolism, and thus has been implicated in the development of osteoarthritis [3,15-18].

Studies have suggested a possible relationship between leptin and OA of weight-bearing joints [15,16]. However, there have been few studies of nonweight-bearing joints - such as the hand joints - to examine nonmechanical risk factors for OA. Leptin was not associated with hand OA progression in a recent study of fewer than 300 subjects [19]. To date, no study has examined the cross-sectional association between leptin and hand OA in a population-based sample. The objective of our study was to determine whether a cross-sectional relationship exists between leptin and OA in a US population-based sample of adults at least 60 years of age.

## Materials and methods

### Study sample

We used data from the Third National Health and Nutrition Examination Survey (NHANES III), a national cross-sectional population-based survey conducted from 1988 to 1994 to assess the health and nutrition of the non-institutionalized US population. Survey data, which include demographics, health status, health disorders, and behaviors among others, were collected by household interviews. Physical examinations and blood draws were also performed by physicians at mobile examination centers. For this analysis we used data from the demographic file [20], the examination file [21], and the serum leptin file [22]. Our analysis was restricted to subjects surveyed from 1991 to 1994 and age 60 years or older because NHANES III conducted the hand examination only on these subjects.

NHANES III subjects were randomly assigned to be tested for leptin [22]. Subjects with rheumatoid arthritis were excluded from the analysis, based on clinical information collected at the physician interview, examination and criteria used by Dillon, Rasch and colleagues [23,24]. Institutional Review Board approval was not necessary because the National Health and Nutrition Examination Survey is a publically available database.

### Data elements

#### Assessment of hand osteoarthritis

We applied a previously developed algorithm for defining hand OA in the National Health and Nutrition Examination Survey. Dillon and colleagues developed a definition of hand OA in the National Health and Nutrition Examination Survey database that is modeled on the American College of Rheumatology definition [23]. The American College of Rheumatology criteria and the NHANES III definition differ slightly, as shown in Table 1[23]. We defined asymptomatic hand OA as cases fulfilling criteria 2 to 4 but not criterion 1. In the group without hand OA, subjects did not have symptoms or physical examination evidence of hand OA. Hand radiographs were not released from NHANES III for analysis.

**Table 1:** Hand osteoarthritis classification criteria

American College of Rheumatology criteria	**Criteria for symptomatic hand OA using NHANES III data adapted from Dillon and colleagues**^**a**^
Hand OA is identified if participants meet criteria 1 to 4:	Hand OA is identified if participants meet criteria 1 to 4:
1. Hand pain, aching, or stiffness	1. Ever had history of hand pain, aching, stiffness lasting more than 6 weeks
2. Hard tissue enlargement of two or more of 10 selected joints	2. At least two sites of hard tissue enlargement, with the combination of first CMC and one other site with Heberden's or Bouchard's nodes
3. Fewer than three swollen metacarpal-phalangeal joints	3. Fewer than three swollen metacarpal-phalangeal joints or participants do not meet the criteria for rheumatoid arthritis
4a. Either hard tissue enlargement of two or more DIP joints, or	4. The presence of bilateral Heberden's nodes at any site
4b. deformity of one out of 10 joints	(would remove this number "5" as this is not one of the criteria used in Dillon's NHANES study. The text was put here to compare with ACR criteria. No such data available from NHANES III

CMC, carpometacarpal; DIP, distal interphalangeal joint; NHANES III, Third National Health and Nutrition Examination Survey. ^a^This is the classification used to define symptomatic hand osteoarthritis (OA) in the present study. Adapted from Dillon and colleagues [23].

#### Leptin

Human studies have shown that leptin levels probably peak between 12 midnight and 2:30 am, and are lowest between noon and early afternoon [25]. In NHANES III, leptin was measured in the morning after an 8-hour overnight fast. Leptin has been shown to remain stable when stored frozen for many years [26-29]. The laboratory analysis for serum leptin was performed by Linco Research, Inc. (St. Louis, MO, USA) using a radioimmunoassay with a rabbit polyclonal antibody against highly purified recombinant human leptin. Detectable concentrations of the assay range from 0.5 to 100 fg/l. Within-assay and between-assay reliability coefficients of variations range from 3.4 to 8.3% for radioimmunoassay and from 3.6 to 6.2% for western blot [22].

#### Potential confounders

We hypothesized that age, sex, race/ethnicity, body mass index (BMI), the presence of polyarticular OA, diabetes, and total cholesterol may confound the relationship between leptin and hand OA. We thus accounted for these variables in the analysis. Age was classified into three groups; 60 to 69 years, 70 to 79 years, and 80+ years. We classified a subject's race/ethnicity as non-Hispanic white, non-Hispanic black, Hispanic, or other. We classified BMI as normal (< 25 kg/m^2^), overweight (25 to 29.99 kg/m^2^), and obese (≥ 30 kg/m^2^). Polyarticular OA was defined as the presence of radiographic knee OA with a Kellgren-Lawrence grade ≥ 2 in at least one knee in addition to having either symptomatic hand OA or asymptomatic hand OA. Total cholesterol was classified as desirable (< 200), borderline high (200 to 239), and high (≥ 240).

### Statistical analysis

To ensure that the sample of subjects with a leptin measurement did not differ from the sample of subjects without a leptin measurement, we compared potential confounders for those with a leptin measurement and for those without a leptin measurement. The remaining analyses were performed on those with a hand examination and a leptin measurement. We calculated percentages by hand OA status for all potential confounders. We estimated unadjusted mean leptin levels by hand OA status and the other potential confounders stratified by sex. We stratified analyses of leptin by sex because the mean and variance of serum leptin concentration was much greater among females than in males. We further applied linear regression to estimate the adjusted mean leptin by hand OA status stratified by sex. To fit the most parsimonious model, we only included potential confounders in the final model if they reached statistical significance at an alpha level of 0.05. We used SAS statistical software (version 9.2; SAS Institute, Inc, Cary, North Carolina, U.S.A.) to perform all analyses. To account for the complex multistage survey design and to obtain estimates representative of the non-institutionalized US population, we used survey procedures within SAS and weighted the analyses using the appropriate survey sampling weight.

## Results

Of the 2,589 subjects who had interviews and hand examinations, examination data were complete in 2,498. Nineteen patients with rheumatoid arthritis were excluded. Of 2,477 subjects that were eligible for the analysis, 1,056 (42.6%) had a serum leptin measurement and comprised our sample. Subjects with leptin measurements and those who did not have leptin measurements were similar with respect to demographic factors (age, sex, race, BMI) (see Additional file 1). Among those in our final sample, hand OA was symptomatic in 90 patients (8.5%), asymptomatic in 376 patients (35.6%), and not present in 590 patients (55.9%).

The mean age of the final sample was 70.3 ± 8.2 years and the mean BMI of the final sample was 26.4 ± 4.9 kg/m^2^. Subjects with hand OA (both symptomatic and asymptomatic) were more likely to be female than those without hand OA. Subjects with symptomatic hand OA were more likely to be overweight (45%) and obese (25%) than those with asymptomatic hand OA (39% overweight and 16% obese). Detailed data on the demographic and clinical characteristics of our sample stratified by hand OA status are shown in Table 2.

**Table 2:** Demographic characteristics of the sample by hand osteoarthritis status

	Symptomatic hand OA	Asymptomatic hand OA	No hand OA
Age			
60 to 69 years	41 (45%)	138 (45%)	309 (58%)
70 to 79 years	35 (46%)	140 (39%)	169 (28%)
80+ years	14 (9%)	98 (17%)	112 (15%)
Sex			
Male	36 (34%)	159 (37%)	315 (49%)
Female	54 (66%)	217 (63%)	275 (51%)
Race/ethnicity			
Non-Hispanic white	47 (85%)	251 (90%)	301 (79%)
Non-Hispanic black	17 (9%)	49 (5%)	124 (8%)
Hispanic	26 (5%)	75 (5%)	153 (9%)
Other	0 (0%)	1 (0%)	12 (4%)
BMI status			
Normal (BMI < 25 kg/m^2^)	22 (31%)	152 (45%)	208 (42%)
Overweight (25 ≤ BMI < 30 kg/m^2^)	39 (45%)	145 (39%)	212 (35%)
Obese (BMI ≥ 30 kg/m^2^)	25 (25%)	50 (16%)	133 (23%)
Radiographic knee OA			
Yes	36 (31%)	130 (32%)	177 (30%)
No	47 (69%)	234 (68%)	391 (70%)
Diabetes			
Yes	19 (15%)	36 (8%)	81 (11%)
No	71 (85%)	339 (92%)	508 (89%)
Total cholesterol			
Desirable (< 200)	27 (26%)	120 (27%)	190 (29%)
Borderline high (200 to 239)	32 (39%)	140 (40%)	218 (39%)
High (≥ 240)	31 (35%)	113 (32%)	173 (32%)

Data presented as *n *(%). Percentages are weighted using Third National Health and Nutrition Examination Survey sampling weights. BMI, body mass index; OA, osteoarthritis.

Unadjusted mean leptin levels stratified by sex and all potential confounders are shown in Table 3. Among males, the unadjusted mean leptin level did not vary substantially by hand OA status. Those with no hand OA had a mean leptin of 8.22 (95% confidence interval (CI) = 7.47, 8.97) compared with 7.38 (95% CI = 5.31, 9.46) for those with symptomatic hand OA and 6.69 (95% CI = 5.19, 8.18) for those with asymptomatic hand OA. Among females, the unadjusted mean leptin did vary slightly by hand OA status, with those with asymptomatic hand OA having the lowest mean leptin at 17.09 (95% CI = 15.00, 19.18). However, all of the 95% CIs overlapped. The mean leptin for females with symptomatic hand OA was 21.55 (95% CI = 17.08, 26.02) and for no hand OA was 20.77 (95% CI = 18.01, 23.53). Mean leptin levels increased as BMI increased for both males and females.

**Table 3:** Unadjusted mean leptin by sex for hand osteoarthritis status and potential confounders in NHANES III

	Male		Female	
	
	*n*	Mean^a ^(95% CI)	*n*	Mean^a ^(95% CI)
Overall	510	7.66 (7.11, 8.21)	546	19.24 (17.19, 21.29)
Hand OA status				
Symptomatic hand OA	36	7.38 (5.31, 9.46)	54	21.55 (17.08, 26.02)
Asymptomatic hand OA	159	6.69 (5.19, 8.18)	217	17.09 (15.00, 19.18)
No hand OA	315	8.22 (7.47, 8.97)	275	20.77 (18.01, 23.53)
Age				
60 to 69 years	255	7.53 (6.83, 8.22)	233	21.43 (18.73, 24.13)
70 to 79 years	147	7.88 (6.47, 9.29)	197	17.60 (14.47, 20.73)
80+ years	108	7.66 (6.24, 9.07)	116	16.00 (12.73, 19.28)
Race/ethnicity				
Non-Hispanic white	273	7.62 (7.06, 8.17)	326	18.73 (16.52, 20.95)
Non-Hispanic black	89	7.30 (5.61, 8.99)	101	24.39 (21.52, 27.25)
Hispanic	139	8.74 (6.24, 11.25)	115	19.34 (13.94, 24.74)
Other	9	7.42 (6.31, 8.53)	4	19.83 (-2.88, 42.54)
BMI status				
Normal (BMI < 25 kg/m^2^)	183	5.00 (4.30, 5.70)	199	11.54 (10.30, 12.77)
Overweight (25 ≤ BMI < 30 kg/m^2^)	217	7.73 (6.75, 8.70)	179	19.65 (17.68, 21.63)
Obese (BMI ≥ 30 kg/m^2^)	90	13.03 (11.03, 15.03)	118	34.55 (31.22, 37.88)
Radiographic knee OA				
Yes	132	8.99 (7.53, 10.45)	211	23.31 (19.55, 27.08)
No	357	7.09 (6.59, 7.60)	315	16.93 (14.99, 18.87)
Diabetes				
Yes	64	8.69 (7.10, 10.27)	72	20.62 (16.47, 24.76)
No	445	7.55 (7.02, 8.08)	473	19.1 (17.13, 21.08)
Total cholesterol				
Desirable (< 200)	201	7.24 (6.33, 8.14)	136	14.87 (12.01, 17.73)
Borderline high (200 to 239)	193	7.36 (6.66, 8.07)	197	18.99 (16.40, 21.58)
High (≥ 240)	113	8.18 (6.92, 9.44)	204	22.20 (18.99, 25.41)

BMI, body mass index; CI, confidence interval; OA, osteoarthritis. ^a^Means are weighted using Third National Health and Nutrition Examination Survey (NHANES III) sampling weights.

Results of the linear regression showed similar findings to the unadjusted analysis. The final model included hand OA status, age, and obesity status. Race/ethnicity, diabetes, polyarticular OA, and total cholesterol were not included in the final model. Among males, the adjusted mean leptin for those with symptomatic hand OA was 8.88 (95% CI = 7.08, 10.67) compared with 8.21 (95% CI = 6.62, 9.80) for those with asymptomatic hand OA and 9.40 (95% CI = 8.59, 10.21) for those with no hand OA. Similarly, among females, the adjusted mean leptin for those with symptomatic hand OA was 21.89 (95% CI = 18.70, 25.07) compared with 20.13 (95% CI = 18.32, 21.95) for those with asymptomatic hand OA and 23.09 (95% CI = 21.83, 24.34) for those with no hand OA (Table 4). None of these differences in adjusted mean leptin levels was statistically significant (all *P *> 0.05).

**Table 4:** Adjusted mean leptin by sex and hand osteoarthritis status

	Male		Female	
	
Hand OA status	**Mean**^**a**^	95% CI	**Mean**^**a**^	95% CI
Symptomatic hand OA	8.88	7.09, 10.67	21.89	18.70, 25.08
Asymptomatic hand OA	8.21	6.62, 9.80	20.13	18.32, 21.94
No hand OA	9.40	8.59, 10.21	23.09	21.84, 24.34

CI, confidence interval; OA, osteoarthritis. ^a^Means are adjusted for age and obesity status, and are weighted using Third National Health and Nutrition Examination Survey sampling weights.

## Discussion

The present study used data from NHANES III to describe the cross-sectional relationship between leptin and hand OA. The study did not provide evidence to support the hypothesis that hand OA is associated with increased serum leptin level. Stratified and multivariable analyses did not show evidence of a difference in leptin levels in the symptomatic hand OA, asymptomatic hand OA, and no hand OA groups. The multivariable analyses adjusted for age, race/ethnicity, BMI status, and the presence of polyarticular OA as indicated by radiographic knee OA, diabetes status, and serum cholesterol.

Our analyses have several important strengths. NHANES III has a large sample size, making it unlikely that we simply missed an effect due to chance. Also, selection bias is unlikely because the NHANES III sample was representative of the US population. Lastly, since we accounted for the complex sample design and used the sampling weights, the estimates are representative of the US population [22].

The study also has limitations. A possible explanation for our null result is that the association between hand OA and leptin is complex and cannot be assessed using a cross-sectional sample. Leptin may be associated with hand OA at one time point of its development only. However, in the longitudinal study carried out by Yusuf and colleagues, serum leptin was not found to be associated with hand OA progression in 6-year follow-up of 248 participants [19]. The paper by Yusuf and colleagues used a longitudinal design with a radiographic case definition [19]. In the present cross-sectional study, we measured hand OA by an algorithm that incorporates symptoms and physical examination. These two approaches are complementary, and neither supports an association between serum leptin and hand OA.

Another explanation for the null result is the possible misclassification of hand OA in NHANES III. There is no gold standard for the diagnosis of hand OA. Our use of the classification criteria suggested by Dillon and colleagues could have resulted in misclassification. Many patients with early or developing OA start with pain, followed by delayed onset of bony enlargement for months or years. Using our criteria, these subjects would be categorized as having no OA. Misclassification of this sort would tend to bias associations toward the null and could indeed play a role in our negative findings.

As expected, our results confirmed that leptin is associated with gender and BMI status, consistent with prior animal and human studies [30-33]. These findings provide face validity for the leptin measurements. Our study is the largest population-based confirmation of such a relationship in humans, with prior studies in the literature having sample sizes ranging from 87 to 426 subjects [30-33].

## Conclusions

We did not find a cross-sectional association between hand OA and serum leptin in our sample. Prospective studies are needed to further evaluate the possibility that elevated leptin levels predate the onset or progression of clinically apparent hand OA.

## Abbreviations

BMI: body mass index; CI: confidence interval; NHANES III: Third National Health and Nutrition Examination Survey; OA: osteoarthritis.

## Competing interests

The authors declare that they have no competing interests.

## Authors' contributions

All authors read and approved the manuscript for publication. MM was involved in the conception and design of the study, analysis and interpretation of the data, drafting the article, and critical revision of the article for important intellectual content. WMR was involved in the analysis and interpretation of data, drafting the article, critical revision of the article for important intellectual content, statistical expertise, and collection and assembly of data. EL was involved in conception and design of the study, analysis and interpretation of the data, critical revision of the article for important intellectual content, and statistical expertise. DHS was involved in the analysis and interpretation of the data and critical revision of the article for important intellectual content. JNK was involved in conception and design of the study, analysis and interpretation of the data, critical revision of the article for important intellectual content, final approval of the article, and takes responsibility for the integrity of the work as a whole, from inception to finished article.

## Supplementary Material

Additional file 1 **table presenting characteristics of the study sample by serum leptin measurement status, comparing demographic, disease, and confounders' characteristics of the sample with leptin measurement with the sample without leptin measurement in NHANES III**. Click here for file
