# Supplementary material for: The relationship between hand osteoarthritis and serum leptin concentration in participants of the Third National Health and Nutrition Examination Survey
Source: Arthritis Res Ther. 2012 May 31;14(3):R132. doi: 10.1186/ar3864 (PMC3446514; doi:10.1186/ar3864)
Supplement: Additional file 1 — table presenting characteristics of the study sample by serum leptin measurement status, comparing demographic, disease, and confounders' characteristics of the sample with leptin measurement with the sample without leptin measurement in NHANES III. [file ar3864-S1.PDF]

Supplementary Table. Characteristics of the study sample by serum leptin measurement status.

	Leptin measured	Leptin not measured
Age		
60-69	488 (52%)	614 (47%)
70-79	344 (33%)	474 (36%)
80+	224 (15%)	333 (16%)
Sex		
Male	510 (43%)	664 (43%)
Female	546 (57%)	757 (57%)
Race/ethnicity		
Non-Hispanic White	599 (83%)	764 (82%)
Non-Hispanic Black	190 (7%)	293 (9%)
Hispanic	254 (7%)	345 (6%)
Other	13 (2%)	19 (3%)
Obesity status		
Normal	382 (42%)	554 (44%)
Overweight	396 (37%)	537 (40%)
Obese	208 (20%)	231 (16%)
Hand OA status		
Symptomatic hand OA	90 (7%)	117 (9%)
Asymptomatic hand OA	376 (39%)	461 (37%)
No hand OA	590 (54%)	843 (54%)
Radiographic knee OA		
Yes	343 (31%)	474 (32%)
No	672 (69%)	816 (68%)
Diabetes		
Yes	136 (10%)	265 (14%)
No	918 (90%)	1,156 (86%)
Total Cholesterol		
Desirable (< 200)	337 (28%)	448 (34%)
Borderline high (200-239)	390 (39%)	488 (34%)
High (≥ 240)	317 (32%)	422 (32%)

*Percentages in table were weighted using NHANES-III sampling weights
